# Psychometric properties of the original and short Hungarian version of the Iowa infant feeding attitude scale

**DOI:** 10.1186/s13006-021-00403-2

**Published:** 2021-07-16

**Authors:** Renáta Ungváry, András Ittzés, Veronika Bóné, Szabolcs Török

**Affiliations:** 1 Faculty of Health and Public Administration, Institute of Mental Health, Semmelweis University, Budapest, Hungary; 2 Department of Applied Statistics, Institute of Mathematics and Basic Science, Hungarian University of Agriculture and Life Sciences, Budapest, Hungary; 3 Department of Methodology for Business Analysis, Faculty of Commerce, Hospitality and Tourism, Budapest Business School, Budapest, Hungary

**Keywords:** IIFAS, Infant feeding, Attitudes, Breastfeeding duration, Hungary, Reliability, Psychometric properties, Short version

## Abstract

**Background:**

The Iowa Infant Feeding Attitude Scale (IIFAS) is a widely used tool to assess attitudes towards infant feeding. Attitudes towards breastfeeding are one of the main influencing factors of feeding choice and breastfeeding duration. Adaptation of the IIFAS to the Hungarian context provides an opportunity for cross-cultural comparisons and helps to target breastfeeding support interventions. The growing number of shortened scales in various fields of research, demonstrates the necessity to adapt to a changing context of data collection to avoid fatigue and dropout among respondents. However, international comparisons are difficult due to the lack of a consensual shortened form of the scale.

The aim of our study was to examine the psychometric properties of the Hungarian version of the IIFAS (IIFAS-H) and propose an 8-item short version that has appropriate construct validity.

**Methods:**

The original IIFAS was translated into Hungarian and then translated back to English. A cross-sectional study based on an internet survey in 2019 was conducted among 553 mothers whose most recent child’s age was between 6 and 36 months. Psychometric properties of the Hungarian IIFAS were determined and compared with international results. In order to obtain a shorter version of the Hungarian scale, we preferably kept those items that are common with other international abbreviated IIFAS versions and deleted items with a corrected item-total correlation or factor loading of less than 0.3, where factor loadings came from a principal component analysis forcing the extraction of one principal component (factor).

**Results:**

The 17-item IIFAS-H showed good psychometric properties with a Cronbach’s alpha of0.73. Further analyses proved that the examined three shortened versions of the IIFAS consisting of 11, 9, and 8 items also showed good properties (Cronbach’s alpha = 0.79, 0.79, 0.76, respectively).

**Conclusions:**

The Hungarian version of the original 17-item long IIFAS proved to be a good measurement tool with good psychometric properties. Based on our analyses, we suggest the use of the 8-item short version (IIFAS-H8) of the scale.

## Background

Recent statistics in Hungary show that the rates of exclusive breastfeeding are significantly lower [1] than recommended by the World Health Organization (i.e., exclusive breastfeeding for 6 months, and then the gradual introduction of complementary foods while continuing breastfeeding for 2 years of age or beyond on demand of the child and the mother) [2]. The majority of mothers (95%) initiate breastfeeding; however, 3–4 days later, just after hospital discharge, the number of exclusively breastfed infants drops below expectations accounting for only 53%. In Hungary’s capital, Budapest, infant formula supplementation happens in the early postpartum days in 40% of cases without medical indication, initiated purely by the mother. Based on the health visitor nurse statistics, the exclusive breastfeeding rate was 35% in 2018 at 6 months of age [3]. However, a new cohort study conducted by the Hungarian Central Statistical Office shows only 17% exclusive breastfeeding at this age [1].

Families in Hungary who need or choose formula for their infant can access it for its full price or purchase it with a medical prescription for half-price during the first 6 months of the child’s life. The official and open database of the Hungarian Health Insurance Provider NEAK reveals a gradual increase over the past years of normal formula amounts prescribed by doctors [4].

It would be important to gather accurate information about the causes of this worldwide tendency and develop a national strategy to improve exclusive breastfeeding rates and overall breastfeeding duration.

According to previous studies [5–8], initiation and duration of breastfeeding are influenced by the following factors: socioeconomic status, education, age, ethnicity, family support, breastfeeding relatives and friends, the value of breastfeeding within the closer community, breastfeeding support in the society, maternity leave, paid maternity leave, breastfeeding protection in the workplace, restriction of formula marketing based on the WHO Code of breast milk substitutes and subsequent resolutions of WHA [9], breastfeeding support in medical facilities, maternal attitude, breastfeeding knowledge, and access to information.

The degree of the influence of these factors on breastfeeding duration is controversial. Since the introduction of Ajzen’s Planned Behavior theory [10], maternal attitude is seen as one of the most significant factors attributable to breastfeeding initiation and duration. Behavioral attitudes comprehend the individual’s perception of the chosen behavior’s positive and negative effects and the expected costs and benefits.

### Measuring attitudes toward breastfeeding

Since 1991, several measuring scales have been developed worldwide to measure breastfeeding attitudes, knowledge, and community support. These were recently collected and analyzed by Corinne Casal et al. [11]. Sixteen measuring devices were found to meet the set criteria. One of the first widely used measures of breastfeeding attitudes with a high predictive value is the *Breastfeeding Attraction Prediction Tool* (BAPT) published in 1992 [12]. The biggest limitation of its applicability is that it consists of 94 items, so the data acquisition takes about 30–35 min. The most commonly used tool for measuring infant feeding attitudes is the *Iowa Infant Feeding Attitude Scale* (*IIFAS)* introduced in 1999 and developed by Arlene de la Mora et al. [13]. Further reviews also confirm that the IIFAS scale is the most useful tool for measuring attitudes towards breastfeeding [14, 15]. The purpose of the 17-item, short question series is to measure attitudes and knowledge about infant nutrition. Using this scale will make it possible to find mothers who may need targeted breastfeeding support.

### Development of the Iowa infant feeding attitude scale

De la Mora and Russel described the instrument’s development and validation process, made the scale available and documented the methodology of assessment [13]. In the course of developing the scale, 26 questions were initially applied aiming at two main topics and to be answered on a five-point Likert scale:

Product dimensions of breastmilk and formula: cost, maternal physical condition, sexual aspects, mental-physical comfort, and nutritional dimensions.

Process dimensions of breastfeeding and formula-feeding: parental role, physical proximity, infant food intake, ease and accessibility of feeding, nocturnal feeding. Items of the final IIFAS scale were selected based on the rank order of 26 attitude questions. The 26-item version was too time-consuming and difficult, particularly for women with lower socioeconomic status and education. The obtainable scores are between 17 and 85 within the scale narrowed to 17 items, where 17 indicates the attitude in favor of formula-feeding. Content and consistency validity has not been examined by de la Mora. However, predictive validity was checked by de la Mora with several data sets, and the results revealed that the measured attitude towards infant feeding predicted the chosen infant feeding method.

### Findings measured with IIFAS in different cultures

Since the scales analyzed in Casal’s meta-analysis [11] are, with a few exceptions, developed within the United States, the question arises as to whether they are suitable for measuring attitudes towards infant feeding in other cultures as well. IIFAS has also been highly prioritized compared to other scales published in the pertaining literature [14–16]. Cronbach’s alpha coefficients were reported ranging between 0.5 and 0.89. Higher attitude scores in all studies correlated with attitudes in favor of breastfeeding and lower attitudes in favor of formula- feeding, while they even predicted or were specifically associated with the choice of feeding in pregnant women.

The scale also proved to be reliable for Hispanic and Latina women in the United States [17]. According to Holbrook et al., Latinas have a higher IIFAS score compared to the average breastfeeding attitude in the United States. This was also reflected in subsequent breastfeeding indicators; however, this high score was not associated with better outcome indicators.

During the validation procedure, the Arabic version of the IIFAS scale performed well, and its predictive value proved to be adequate [18]. The data extracted from the sample of Lebanese women produced results that favored essentially formula nutrition. The IIFAS score correlated solely with the number of breastfed children. The examined sample also showed a correlation already proven by other investigators that higher maternal age reduces breastfeeding rates, contrary to the tendencies observed in the United States and Europe.

The Japanese scale was also prepared by two independent research groups [19, 20], but only its second version was back-translated, and only then was it detected that some relevant nuances of the translation had been inadequate. The results show that while Japanese women acknowledge the benefits of breastfeeding, overall they prefer formula feeding and find it more appropriate.

The mainland Chinese study of Dai et al. found the measure to be useful, although they note that the Cronbach’s alpha value is only 0.623. While this is acceptable, it suggests that the measure should be adapted to the Chinese cultural environment [21].

Comparably good psychometric indices were measured in Ethiopia (Cronbach’s alpha = 0.79). Ethiopian culture strongly favors breastfeeding, so the higher score on the IIFAS scale showed strong predictability for breastfeeding beyond 24 months [22].

Lau et al. used the scale in the multi-ethnic environment of Singapore [23]. They found that Malay and Chinese women had a less positive attitude towards breastfeeding than Indian women, even though surveys indicate that Indian and Chinese mothers tend to start with breastfeeding and breastfeed longer than other ethnic groups. The authors did not find an explanation for this culture-based discrepancy.

Scott and colleagues investigated expectant couples’ infant feeding attitudes in Glasgow and its association with postnatal feeding [24]. As measured by IIFAS, maternal feeding attitudes showed a stronger correlation with the chosen feeding method than socioeconomic parameters. Parental attitudes of formula-feeding couples did not differ, but IIFAS scores of breastfeeding mothers were higher than their partners.

Scott and colleagues also applied IIFAS during their interviews in four European countries: Sweden, Italy, Spain, and Scotland [25]. The largest difference between the responses was given during the assessment of item eight (public breastfeeding). The researchers concluded that the duration of breastfeeding is more significantly influenced by country-specific social norms than maternal attitudes. Long breastfeeding periods in Sweden were associated with the widespread acceptance of public breastfeeding, while in Italy and Scotland, low acceptance of public breastfeeding was associated with very short breastfeeding periods.

Wallis et al.’s study was the first validation process of the IIFAS scale in Eastern-Europe [26]. The Romanian IIFAS scale shows that some aspects of infant feeding attitude change over time. Expectant mothers were more dismissive towards breastfeeding in public than postpartum mothers. IIFAS-R scores were in the neutral range in both groups, and in comparison to other research, the internal consistency was relatively low, with Cronbach’s alpha 0.50 in the expectant mothers’ group and 0.63 in the maternity group.

Sittlington et al. used an English-language IIFAS scale in their research in Northern Ireland [27]. At the same time, they simplified the clause of the first item in the original scale, that is, ‘The nutritional benefits of breastmilk last only until the baby is weaned from breastmilk.’ Thus, the Northern Irish version of the item was altered (i.e., ‘The benefits of breastmilk last only as long as the baby is fed’). As a result, it can be stated that content simplifications make the question easier to answer even in lower-educated populations, thereby increasing the reliability of the scale.

In a Croatian study [28], a questionnaire measuring breastfeeding knowledge and attitudes was completed by healthcare workers as participants of the WHO UNICEF Baby-Friendly Hospital course, before enrollment and 3 months after its completion, in order to determine the impact of the course on participants’ attitudes and knowledge. The IIFAS scale was used to measure the participants’ attitudes towards breastfeeding. The IIFAS scale was also used to measure the breastfeeding attitudes of nurses working in NICU [29], with an average score of 69, which was interpreted as a positive attitude toward breastfeeding. Thus, the IIFAS scale, in line with the findings of other scientific investigations, is not only suitable for assessing the infant feeding attitudes of pregnant women and mothers but can also be used for this purpose with professionals and volunteer helpers.

### Shortened versions of the IIFAS

Subsequently, shortened versions of several carefully developed measuring instruments were published. The more complex scales are generally considered more reliable [30]. They cover the examined construct in more detail and decrease measuring errors. However, research indicates that longer questionnaires are more likely to bore and frustrate participants and result in incomplete responses [31]. In contrast, shortened scales have the necessary validity and reliability, and at the same time, they decrease dropout due to fatigue [32]. However, in some cases, the need for shortening a scale may occur due to cultural differences. Certain elements of the scale are not relevant in a particular culture [16]. Therefore, dropping those elements increases the scale’s internal validity in that environment.

Studies have reported conflicting evidence on the reliability of the IIFAS scale. Cronbach’s alphas ranged between 0.50 and 0.89, but, as Tomás-Almarcha noted [33], this was not explained. This encouraged the search for solutions with better reliability. One possible solution is to eliminate items that have a low corrected item-total correlation (CITC) if the Cronbach’s alpha is higher as a result. Statistical analyses focused on items that influenced the internal consistency and predictive validity negatively while the corrected item-total correlation was low.

Nanishi et al.’s longitudinal sample [20] found that the IIFAS-J score was not significantly associated with exclusive breastfeeding rates at 4 and 12 weeks postpartum. Surprisingly, the results of this study may stem not only from possible errors of translation but also from diverse cultural circumstances and different ways of thinking, as mentioned above.

AlKusayer et al. [34] decided that special knowledge is required to answer questions 4 and 17. For the item enquiring about alcohol consumption, the corrected item-total correlation was found to be low in several other studies. In the case of item 16, the respondents’ agreement was almost unanimous; therefore, this item did not prove suitable for differentiation between different respondent attitudes. For item 11, the responses did not affect the breastfeeding intention and mode of infant feeding.

Lau et al. [23] examined the usability of the scale in an Asian multi-ethnic context. Consistent with other studies on Asian samples, the researchers omitted item 17 concerning alcohol consumption and the statement regarding overfeeding formula-fed babies.

Ghasemi et al. [35] left out six items since the majority of Iranian society is Muslim, alcohol consumption and public breastfeeding are irrelevant for the sample. The other four items specifically connected to breastfeeding information did not show a high CITC value due to Iranian women’s lack of knowledge regarding breastfeeding. Not all studies adapting IIFAS aimed to develop a shortened scale that was better suited to their own cultural environment; however, several named those items with a low (less than 0.3) corrected item-total correlation. In the Ethiopian sample [22], answers indicate a lack of knowledge regarding iron intake and the irrelevance of men’s role in breastfeeding. Furthermore, minor wording adjustments were needed. There are no restaurants in most of the country, and therefore for public breastfeeding, the words were changed to ‘wedding places and marketplaces’.

In the Arabic version of the IIFAS scale [18], the behavior of items 8 and 17 can be well explained by the mostly typical attitudes of Arabic cultures toward alcohol consumption and public statements of intimacy.

Table 1 provides an overview of the currently available short IIFAS versions and their main characteristics.

**Table 1:** Shortened versions of the IIFAS-scale

Research	Study design	Cronbach’s alpha of the 17-item scale	Mean	Number of items	Left out items’ numbers	Cronbach’s alpha of the shortened scale
Tomás-Almarcha 2016, Spain IIFAS-S9 [33]	Convenience sample 1354 pregnant women, prospective	0.72	66.12	9 items	1, 4, 5, 8, 10, 11, 16, 17	0.79
AlKusayer 2018, Canada IIFAS-C13 [34]	Cross-sectional, 1238 pregnant women	0.87		13 items	4, 11, 16, 17	0.86
Nanishi 2014, Japan IIFAS-J16 [20]	Longitudinal, 781 pregnant women	0.46 (citing Inoue [19])	61.04	16 items	17	0.66
Ghasemi 2018, Iran IIFAS-I11 [35]	Cross-sectional, 280 breastfeeding mothers	not reported	neutral (49–69)	11 items	1, 4, 5, 8, 10, 17	0.85
Ying Lau Singapore 2016 [23]	Cross-sectional, 417 multi-ethnic, English speaking pregnant women	not reported	different between ethnic groups, 62–57	5, 17		0.79
Further research results indicating low CITC for certain items:						
Abdulahi 2020, Ethiopia [22]	Cross-sectional, 468 pregnant women	0.72	65.7		4, 11	
Charafeddine 2016 IIFAS-A Lebanon [18]	Cross-sectional, convenience sample, 196 Arab women, pregnant or peer counselors	0.64	37–85, 72.4% neutral (60–75) attitude		8, 17	0.693
Dai 2013, China [21]	Convenience sample of 660 postpartum women, prospective	0.623	59.82		6, 10, 17	0.76

### Factorial structure

De la Mora et al. did not examine the underlying factors in the process of developing the IIAFS scale; however, based on the meaning of the scale items, analysts assumed two factors behind the scale values: attitudes toward breastfeeding and attitudes toward formula-feeding [13].

Since the publication of IIFAS, several analyses have attempted to explore the internal structure of the IIFAS-scale. Both the exploratory and confirmatory factor analyses have found different factor numbers in different studies.

Some studies aimed to adapt and shorten the original IIFAS-scale to create a one-factor shortened version. Tomás-Almarcha [33] in Spain and Nanishi [20] in Japan created 9- and 16-item shortened versions, respectively, following a principal component analysis (PCA). Other researchers assumed that underlying the IIFAS results are culture-specific factorial structures. In Iran, Ghasemi [35] utilized confirmatory factor analysis to prove that underlying the Iranian sample are two factors. The authors identified these as positive attitudes towards breastfeeding and positive attitudes towards formula-feeding. Several studies, such as AlKusayer’s in Canada [34] or Lau’s in Singapore [23], also identified these two factors employing the commonly used abbreviations: FBF (favor to breastfeeding) and FTF (favor to formula-feeding). In addition, the exploratory factor analysis (EFA) showed an additional eigenvalue greater than 1 for one factor, called convenience (CON). Analyzing the Chinese sample, Dai et al. [21] also identified these three factors and an additional one using the same method (EFA). They named this fourth one sociological influence.

The factorial structures discovered do not differ only in their number of factors. Of the 17 items of the Canadian sample, 7 belong to FBF, 5 to CON, and 5 to FTF. In contrast, in the 15-item Singapore version, these numbers are 6, 7, and 3 items. In the Chinese sample, the distribution is 8, 6, and 2 (supplemented by 2 belonging to social influence – in the analysis, several items demonstrated a relatively high loading on two factors).

According to Lau et al.’s research in Singapore [23], one possible explanation is that differences in factor structure may be based on different perceptions and attitudes towards breastfeeding. This can further explain why the construct validity is so low in some research papers and the Japanese sample. These explanations are further established by the Value theory of Hofstede [36]: as a shared national heritage is “an important determinant of cultural similarity.” Although breastfeeding is a global, female/maternal experience worldwide, there are many differences in details and attitudes between cultures and nations.

## Methods

### Aim

The aim of our research was to examine the psychometric properties of the Hungarian version of the Iowa Infant Feeding Attitude Scale and to make a recommendation for a shorter version of the scale for further research purposes. We examined the relationship of the results of the scale with the mothers’ infants feeding status at 4 and 6 months of age in our sample.

### Design

Data collection was realized with the aid of an online internet survey provider named *kerdoivem.hu*. After adding items aiming to extract the respondents’ demographic data and breastfeeding attitudes to the scale, the measure was used for an online survey between the 16th of April and the 2nd of May 2019. Our sample proved to be a convenience sample since the invitation for participation was circulated during this period of time on mother-baby group mailing lists, on social media, in thematic groups concerned with breastfeeding support, baby-nutrition, and mothers who gave birth with cesarean section. The questionnaire was supplemented with a brief summary of the research objectives and conditions of participation. The questionnaire was only allowed to be completed once from the same IP address. The completion of the scale was anonymous, and respondents were informed about the electronic storage of their data in compliance with the regulations pertaining to personal data storage.

Taking into account aspects related to the respondents (i.e., the respondent must be older than 18 but under 49 years of age, have a child between 6 and 36 months of age, and have a residence in Hungary), out of 638 respondents 554 remained, and finally, we analyzed only the responses of women out of the responses of 553 women and one man. Limitations that originated from the internet survey method are further discussed in the limitations part of this paper.

### Measures

Two authors of this article individually translated the original 17-item IIFAS into Hungarian in 2018. After their consensus, an independent, bilingual translator who was not familiar with the instrument translated it back to English. Since the back translation and the original version had shown no significant differences, the wording of the Hungarian scale was finalized.

The IIFAS scale is composed of 17 items that are rated on a 5-point Likert scale (ranging from 1 = strongly disagree – 5 = strongly agree). Eight items are favorable towards breastfeeding and nine items towards formula-feeding that are reverse coded. The total score ranges from 17 to 85, where the lower score indicates a more positive attitude towards formula-feeding.

Participants completed the 17-item IIFAS scale and were asked to provide demographic data such as income, residence, education, and infant feeding status at different ages (1, 4, and 6 months of age) of their youngest child aged 6–36 months as suggested by de La Mora [13]. We measured infant feeding status with the following question:

*‘How were you feeding the baby at 1 (4, 6) months of age?’* Answers were on a 5-point scale:

1: exclusive breastfeeding; 2: breastfeeding with little formula supplementation; 3: approximately half breastfeeding, half formula-feeding; 4: mainly formula, little breastfeeding; or 5: exclusive formula-feeding.

### Characteristics of participants

Due to the convenience sampling method we used, volunteer bias may have occurred in our results. The participants were recruited via thematic online groups or social media where the appearance of mothers with higher education, more consciousness in child-rearing, and positive attitudes towards breastfeeding can be anticipated. In this group, a greater motivation for answering questions and sharing opinions can be prevalent [37, 38]. The research population consisted of mothers who had a child between 6 and 36 months of age. Since there are no available databases of the mothers of the given children population in Hungary, we compared our respondents’ demographic data to the Cohort’18 Growing Up in Hungary [39] and the Infancy in twenty-first Century Hungary [40] studies. The Cohort’18 countrywide longitudinal birth cohort investigation is conducted by the Hungarian Demographic Research Institute and follows participants (*n* = 8409) from before birth. The Infancy in twenty-first Century project collected data from parents (*n* = 980) who had a child between 3 and 36 months of age in 2020. The Cohort ‘18 study was representative for the districts in which the children live. The Infancy in the twenty-first Century Hungary study was representative for the children’s age, gender, and type of residence. Both of them were recruited cohorts. Breastfeeding data were collected in both representative studies. In the Cohort’18 study, exclusive breastfeeding rate at 6 months of age is 17%. Breastfeeding data from the Infancy in twenty-first Century Hungary study are not yet public.

Descriptive characteristics of the sample and comparisons with related samples of the above-mentioned representative research in Hungary are shown in detail in Table 2. The 553 participant women ranged in age from 21 to 47 years (M = 32.88 years, SD = 4.84), which means our respondents are younger than the national average of mothers. The most remarkable difference can be observed in education level. Our sample consists of mostly well-educated women: 70% of participants had completed at least college or higher education. Mothers with fewer children were more likely to fill out the questionnaire: 63% of participants were first-time mothers, 23.9% had two children, and 12.3% had three or more children. On the basis of monthly family income, 38.3% of participants had low income, 37.4% had a middle-range income, and 24.2% had a high income. About one in five (22.8%) of participants were currently working; 64.7% of mothers were breastfeeding their youngest child at the time of questionnaire completion; 70% of mothers were exclusively breastfeeding when the youngest child was 6 months old. This indicates that in our sample, breastfeeding mothers are overrepresented. Current Hungarian research results [1] and data [3] show a much lower breastfeeding rate, such as between 17 and 35% for exclusive breastfeeding at 6 months.

**Table 2:** Sociodemographic characteristics of the sample

	*n* (%) Present study	*(%)* Cohort ‘18	*%* Infancy in twenty-first C. H.
Maternal age			
21–29 years	150 (27.1)	46.1 (−29 years)	54.1 (18–30 years)
30–39 years	350 (63.3)	48.5 (30–39 years)	42.8 (31–40)
40–47 years	53 (9.6)	5.4 (40- years)	3.1 (41–52)
Parity			
one child	349 (63.1)	46.5	62.9
two children	132 (23.9)	32.9	24.1
more than two children	68 (12.3)	20.6	8.7
Level of education			
primary	20 (3.6)	20.1	17.8
secondary	107 (19.3)	45.3	70.4
university	426 (77.0)	34.6	18
Monthly income			
low income	212 (38.3)	N.D.	N.D.
middle range income	207 (37.4)		
high income	134 (24.2)		

### Data analysis

All statistical analyses were performed using the IBM SPSS (Statistical Product and Service Solutions) for Windows, Version 25. Descriptive analyses of the participants’ sociodemographic characteristics and the IIFAS items’ scores, and the IIFAS scale were carried out. The correlation between the infant feeding status at 4 and 6 months of age and the IIFAS scores were evaluated by Spearman’s *rho* (rank correlation).

Reliability of the translated 17-item IIFAS and the shortened scales were assessed using Cronbach’s *alpha* coefficient, estimation of *alpha* when an item was deleted from the scale, and corrected item-total correlations.

In order to obtain a shorter version of the scale, we deleted items with a CITC or factor loading of less than 0.3, where factor loadings came from a principal component analysis forcing the extraction of one principal component (factor).

Hierarchical logistic regression analyses were conducted to evaluate whether attitudes towards infant feeding could influence the actual type of feeding method when the infant was 6 months old, over and above the effects of sociodemographic variables such as maternal age, educational level, and the number of biological children. The dependent variables of the analyses were the binary variables of exclusive breastfeeding at the given date. Significance of the models, Nagelkerke’s R^2^ and odds ratios (OR) were calculated.

## Results

### The Hungarian version of IIFAS with the original 17 items

The 17-item IIFAS scores of the 553 respondents ranged from 37 to 85, with a mean of 66.76 and a standard deviation of 9.00.

The Cronbach’s *alpha* coefficient of the IIFAS-H was 0.73. Table 3 shows descriptive statistics (mean and standard deviation), the CITCs and Cronbach’s *alpha* values when an item was deleted. The last column of this table shows the factor loadings of the first principal component.

**Table 3:** Basic statistics and main results of reliability analysis and principal component analysis forcing one factor for the IIFAS-H (*n* = 553)

	Mean	St. Dev.	Corrected Item-Total Correlation	Cronbach’s Alpha if Item Deleted	Factor Loading
1.^*^ The nutritional benefits of breast milk last only until the baby is weaned from breast milk.	2.92	1.76	0.05	0.76	0.07
2.^*^ Formula-feeding is more convenient than breastfeeding.	4.17	1.25	0.37	0.72	0.45
3. Breastfeeding increases mother-infant bonding.	4.57	0.91	0.51	0.71	0.64
4.^*^ Breast milk is lacking in iron.	3.83	1.11	0.14	0.74	0.16
5. Formula-fed babies are more likely to be overfed than are breastfed babies.	3.50	1.44	0.46	0.71	0.63
6.^*^ Formula-feeding is the better choice if a mother plans to work outside the home.	3.62	1.29	0.37	0.72	0.45
7. Mothers who formula-feed miss one of the great joys of motherhood.	2.71	1.45	0.43	0.71	0.60
8. Women should not breastfeed in public places such as restaurants.	4.36	1.07	0.33	0.72	0.40
9. Babies fed breast milk are healthier than babies who are fed formula.	3.42	1.40	0.49	0.70	0.68
10.^*^ Breastfed babies are more likely to be overfed than formula-fed babies.	4.55	0.84	0.30	0.72	0.37
11.^*^ Fathers feel left out if a mother breastfeeds.	4.07	1.16	−0.004	0.75	−0.02
12. Breast milk is the ideal food for babies.	4.92	0.42	0.22	0.73	0.33
13. Breast milk is more easily digested than formula.	4.44	1.00	0.46	0.71	0.63
14.^*^ Formula is as healthy for an infant as breast milk.	3.67	1.27	0.54	0.70	0.68
15. Breastfeeding is more convenient than formula-feeding.	4.08	1.23	0.50	0.70	0.60
16. Breast milk is less expensive than formula.	4.65	0.91	0.16	0.73	0.23
17.^*^ A mother who occasionally drinks alcohol should not breastfeed her baby.	3.26	1.50	0.28	0.73	0.38

^*^Items marked with asterisks are reverse-scored to show a positive attitude toward breastfeeding

Due to low factor loadings (less than 0.3 in Table 3), four items (items 1, 4, 11 and 16) can be considered problematic. For these four and two other items (items 12 and 17), the CITC is also under 0.3.

Hierarchical logistic regression was carried out with the dependent variable of exclusive breastfeeding when the infant was 6 months old (1 = yes, 0 = no). The first block of the three sociodemographic variables gave a significant model (chi^2^(6) = 19.8, *p* < 0.01) but only the number of biological children was significant (OR = 1.46, *p* = 0.01), and the explanatory power of the model was low (Nagelkerke’s R^2^ = 0.05). After entering the IIFAS score, the model substantially improved. Both the difference of the models (chi^2^(1) = 159.7, *p* < 0.01) and the second model (chi^2^(7) = 179.5, *p <* 0.01) were significant. The OR of the number of biological children was weaker than in the first model (OR = 1.35, *p* = 0.09), but the OR of the IIFAS score was significant (OR = 1.18, *p <* 0.01). The explanatory power of the model became much higher (Nagelkerke’s R^2^ = 0.39).

We conducted a similar hierarchical logistic regression analysis with the dependent variable ‘exclusive breastfeeding at four months of age’, and the results, in general, were very similar to the first model with the 6-month-focused data.

These results show that over and above the sociodemographic variables, the IIFAS score could provide important information on predicting whether a mother will breastfeed her child exclusively at 4 or 6 months of age.

### Possible shortened forms of the Hungarian version of IIFAS

If we expect a scale in which each item should have a CITC and factor loading in the first principal component of at least 0.3, then we get a shortened form of the Hungarian IIFAS, which consists of 11 items. As mentioned above, items 1, 4, 11, 12, 16, and 17 must be deleted since these six items have a CITC less than 0.3. In addition, four of them should be deleted because of the low factor loadings as well.

This 11-item version of the scale has better characteristics than the 17-item scale (e.g., its Cronbach’s alpha is 0.79 and all the factor loadings of the first principal component are greater than 0.3), but the CITC of two items (item 8 and 10) are a little under 0.3 (0.29). If we use the rule mentioned earlier, these items have to be deleted, and we get a 9-item version of IIFAS. If we compare the items of this version with other shortened forms of IIFAS from different cultures and languages (Canadian 13-item version, Iranian 11-item version, Spanish 9-item version), we can declare that eight items are common in these four shortened forms. The additional item of the Hungarian 9-item version includes the statement about overfeeding (‘Breastfed babies are more likely to be overfed than formula-fed babies.’). Table 4 gives an overview of the items of the different shortened forms.

**Table 4:** Recommended shortened forms of IIFAS in different cultures based on the literature and this study

	Canadian	Iranian	Spanish	Hungarian (11 items)	Hungarian (9 items)
1.^*^ The nutritional benefits of breast milk last only until the baby is weaned from breast milk.	yes	no	no	no	no
2.^*^ **Formula-feeding is more convenient than breastfeeding.**	**yes**	**yes**	**yes**	**yes**	**yes**
**3. Breastfeeding increases mother-infant bonding.**	**yes**	**yes**	**yes**	**yes**	**yes**
4.^*^ Breast milk is lacking in iron.	no	no	no	no	no
5. Formula-fed babies are more likely to be overfed than are breastfed babies.	yes	no	no	yes	yes
6.^*^ **Formula-feeding is the better choice if a mother plans to work outside the home.**	**yes**	**yes**	**yes**	**yes**	**yes**
**7. Mothers who formula-feed miss one of the great joys of motherhood.**	**yes**	**yes**	**yes**	**yes**	**yes**
8. Women should not breastfeed in public places such as restaurants.	yes	no	no	yes	no
**9. Babies fed breast milk are healthier than babies who are fed formula.**	**yes**	**yes**	**yes**	**yes**	**yes**
10.^*^ Breastfed babies are more likely to be overfed than formula-fed babies.	yes	no	no	yes	no
11.^*^ Fathers feel left out if a mother breastfeeds.	no	yes	no	no	no
12. Breast milk is the ideal food for babies.	yes	yes	yes	no	no
**13. Breast milk is more easily digested than formula.**	yes	yes	yes	yes	yes
14.^*^ **Formula is as healthy for an infant as breast milk.**	yes	yes	yes	yes	yes
**15. Breastfeeding is more convenient than formula-feeding.**	yes	yes	yes	yes	yes
16. Breast milk is less expensive than formula.	no	yes	no	no	no
17.^*^ A mother who occasionally drinks alcohol should not breastfeed her baby.	no	no	no	no	no

^*^Items marked with asterisks are reverse-scored to show a positive attitude toward breastfeeding

Only items marked with ‘yes’ are part of the given shortened scale

Items marked with ‘no’ are not part of the given shortened version

**Bold:** the 8 items of the recommended IIFAS-H8

All the analyses used to investigate the 17-item Hungarian version of IIFAS were also run for each of the 11-item, the 9-item, and the 8-item scales. For the 9-item and 8-item scales, all the CITCs and factor loadings are already above 0.3 (and what is more, the minimum values of factor loadings were 0.41 and 0.47, respectively). All the other statistical parameters of these scales were practically as good as those of the 17-item version. The most important statistical results are summarized in Table 5.

**Table 5:** Some statistical results when analyzing the original and the three shortened forms of the Hungarian version of IIFAS (*n* = 553)

	Hungarian versions of IIFAS			
	17 items	11 items	9 items	8 items
Cronbach’s alpha	0.73	0.79	0.79	0.76
Correlation^a^ of IIFAS score with…				
feeding status at 4 months of age	0.47 (*p <* 0.01)	0.49 (*p <* 0.01)	0.49 (*p <* 0.01)	0.47 (*p <* 0.01)
feeding status at 6 months of age	0.55 (*p <* 0.01)	0.56 (*p <* 0.01)	0.55 (*p* < 0.01)	0.52 (*p <* 0.01)
Results of hierarchical logistic regression if the dependent variable is exclusive breastfeeding at 6 months of age				
Nagelkerke’s R^2^ of the first model^b^	0.05	0.05	0.05	0.05
Nagelkerke’s R^2^ of the second model^c^	0.39	0.44	0.44	0.41
OR of IIFAS score in the second model^c^	1.18	1.24	1.26	1.28

^a^Spearman’s rank correlation

^b^Using maternal age, maternal education level, and number of biological children as predictors

^c^Using maternal age, maternal education level, number of biological children, and IIFAS score as predictors

Although the convenience sample method using an internet survey excludes the possibility of confirming the predictive validity of the scale, we found it informative and useful to test the correlation between IIFAS scores and breastfeeding performance at 4 and 6 months of age of the infants of the mothers as well.

## Discussion

Our first aim was to assess the usability of the IIFAS scale in the Hungarian context. Previous studies measured different psychometric properties in different cultures during scale validation. The 0.73 Cronbach’s alpha, confirming the internal validity of the 17-item full scale, indicates that the longer, full version of the scale can be utilized in future studies. Some CITC values of the 17 items have different values, similar to other adaptations. Thus, it is possible that in addition to a similar or slightly lower but acceptable Cronbach’s alpha level, a shorter Hungarian questionnaire may measure attitudes towards infant feeding well.

Our results revealed a low CITC value of the following items: 1, 4, 11, 12, 16, and 17. We omitted them, and the 11-item shortened Hungarian version demonstrated a high level of construct validity, having a Cronbach’s alpha of 0.79. At the same time, one of the main goals of adapting scales, namely, to make the same constructs measured in different cultures comparable, is impeded by the different results produced by shortened versions. We checked which elements had lower CITC values (around or below 0.3) in several studies – whether the authors omitted them from the final version or not. We found eight items that were included in most of the shortened scales regardless of cultural context. In the Hungarian sample, the Cronbach’s alpha of these items is 0.76. Although this is lower than the 11-item value, this disadvantage is more than offset by the possibility of future international comparisons.

When evaluating omitted items, we must note that even the authors of the IIFAS scale [13] ponder the theoretical question of whether the scale really does measure attitudes or rather just beliefs. For instance, in the case of question 4 (‘Breast milk is lacking in iron’), the vast majority of respondents are not in possession of the knowledge of the amount of iron measured in breast milk and whether it is present at all. Furthermore, the expression used in the questionnaire of *lacking in iron* makes mothers more insecure in relation to this matter since they would require factual figures as a benchmark for their balanced answer. However, if we measure beliefs, a respondent who favors breastfeeding will choose the option of ‘does not agree at all’ because, in line with his or her attitude, breast milk should not lack such an important micronutrient. If, on the other hand, we consider it as a knowledge-testing question, the neutral answers will dominate because the respondent simply does not know how much iron is in breast milk.

The same is true of question 1 (‘The nutritional benefits of breast milk last only until the baby is weaned from breast milk.’): it is not only individuals with a lower level of education who will find it difficult to interpret the expression of “nutritional benefits.” As indicated by the typically low CITC of these questions, this issue generates insecurity even in those respondents who strongly favor breastfeeding. This might be one reason why the simplified version of this question has been used in Northern Ireland research [27], asking simply about the long-term benefits of breastfeeding. No matter whether we measure knowledge, attitudes, or beliefs with these two questions, they do not align with the other items on the scale. Therefore, items 1 and 4 were also left out when we were looking for briefer options to make the scale easy to use in other research because of its brevity, clarity, and more accurate measurement results.

Questions 8 (public places) and 17 (‘A mother who occasionally consumes alcohol should not breastfeed her baby.’) are strongly related to social environments and their norms, as revealed by a survey of several national versions of IIFAS [18, 20, 24, 35, 41]. In the case of question 17, the answer is not primarily determined by the level of knowledge about breastfeeding, nor by the attitude towards breastfeeding, but rather by social acceptance of alcohol consumption, including maternal alcohol consumption. This question is most likely not suitable for measuring attitudes towards infant feeding in its present form.

Item 8 examines attitudes towards breastfeeding in public venues. Our survey results revealed that the vast majority of respondents, irrespective of the feeding method used, do not agree that mothers should not breastfeed in public places. Slightly more than 80% of respondents support public breastfeeding, with only 7.8% clearly dissenting and 11.9% taking a neutral position. However, the recognition of the right to breastfeed in public does not mean that this option is fully accepted or desired by the respondent as well. Wallis’s research [26] in Romania – that asked pregnant women and postpartum mothers – revealed that the consideration of the issue is different for a respondent without a child or when the child is being expected, from the one when the baby is already born, and the acceptance of public breastfeeding also provides more external room for the mother to move around with her infant. In the first 11-item version of the IIFAS-H, the CITC of item 8 was just slightly below the limit. This location can be explained by the fact that personal experiences and previous experiences count equally besides attitudes when judging the issue. Therefore, this question, in addition to asking a very important point, is less suitable for measuring infant feeding attitudes and predicting behavior in this form, similar to Alkusayer’s finding [34]. Therefore, we left this item out of the short Hungarian version, as well.

In the case of questions 12 (‘Breast milk is the ideal food for babies.’) and 16 (‘Breast milk is less expensive than formula.’), the CITC is likely to be low for different reasons than the ones already mentioned above. This is because these two items contain generally accepted statements to which few respondents have any objections, and even the majority of respondents in favor of formula nutrition agree with their content. Therefore, these questions are less suitable for differentiating between the two extreme infant feeding behaviors compared to other items.

For question 11 (‘Fathers feel left out if a mother breastfeeds’), the distribution of responses in our sample resulted in an extremely low CITC. The desire for fathers to strive for equal participation in upbringing and care assures this item *raison d’être*, as fathers are traditionally excluded from breastfeeding an infant. Due to globalization trends, there is a growing expectation for fathers to get involved in the personal care of the baby and infant feeding in Western countries. However, based on the data from our respondents, we can state that the vast majority, that is, 71.5% of the respondents, deny that fathers miss out on childcare because of breastfeeding. In our sample, this percentage corresponds approximately to the proportion of mothers breastfeeding exclusively for 5 months. In Hungary, the development of gender roles within the family follows a traditional pattern [42]; however, maternity leave can be considered long compared to worldwide trends (24 weeks). In addition, the authorized maximum period of time spent at home with the child is 2 years, which is funded by 70% of the parent’s salary. Childcare is considered to be primarily the duty of the mother by society. This explains why a low CITC in our study indicates that the majority of respondents reject the idea that fathers should feel obliged to get involved in infant feeding as well.

### Limitations

Our data were collected using an online survey. According to principles described in Ball’s paper [43], the use of social media and virtual communities to distribute invitations to a survey can lead to sample bias. We cannot access any response rate because we do not know how many people saw our survey announcement. In our sample, mothers with higher education degrees and longer overall breastfeeding durations were overrepresented; however, this tendency is present in most IIFAS related papers working with convenience samples. In our sample, people with low socioeconomic status were strongly underrepresented. This leads to volunteer bias, as described in the paragraphs discussing demographic characteristics of our research sample in the methods section. Due to anonymity, survey fraud cannot be ruled out. Breastfeeding data were collected retrospectively and self-reported, so they are probably less accurate. The survey was conducted on a convenience sample of motivated volunteers. This can lead to non-response bias that occurs due to systematic differences between responders and non-responders. A selection bias could also have occurred due to the fact that our internet survey was conducted in thematic social media groups that differ systematically from the population of interest. A volunteer bias could also have occurred because mothers who volunteered to participate in our survey most likely had different characteristics from the general population [38].

The convenience sample method using an internet survey excludes the possibility of confirming the scale’s predictive validity. Also, this sampling is not unprecedented in research with the purpose of scale validating. Further research is needed on a representative sample to assess and explain connections between breastfeeding duration, demographics, and IFFAS scores.

## Conclusion

In particular, it was necessary to examine the psychometric characteristics of IIFAS-H in order to develop a tool for measuring attitudes towards infant feeding that could be applied in other research. In addition to the original 17-item version, the 11-item and 9-item versions also proved to be reliable. Compared to the international abbreviated IIFAS versions, we found eight items that are common across each version, and at the same time, are part of our Hungarian 9- and 11-item scales, too. The Cronbach’s alpha calculated for these eight items also confirmed their high internal consistency. In summary, the reliability of each of the abbreviated scale versions is better than the original version consisting of 17-items. The reliability of the 8-point scale (IIFAS-H8) is only slightly lower than that of the 9-item scale (Cronbach’s alpha = 0.76 vs. 0.79). When adapting any scale, the best possible comparability of the international results is a high priority; therefore, we recommend the use of the 8-point scale in the future.

## Data Availability

The datasets used and analyzed during the current study are available from the corresponding author on reasonable request.

## Abbreviations

BF: Breastfeeding
CITC: Corrected item-total correlation
CON: Convenience
EFA: Exploratory factor analysis
F: Formula
FBF: Favor to breastfeeding
FF: Formula feeding
FTF: Favor to formula feeding
IIFAS: Iowa infant feeding attitude scale
NICU: Neonatal intensive care unit
SES: Socioeconomic status
